# Celebrating the 100th anniversary of Sun Yat-Sen University

**DOI:** 10.1039/d5sc90062j

**Published:** 2025-03-12

**Authors:** Wei-Xiong Zhang, Cheng-Yong Su, Xiao-Ming Chen, Song Gao

**Affiliations:** a School of Chemistry, IGCME, Sun Yat-Sen University Guangzhou 510275 China zhangwx6@mail.sysu.edu.cn cesscy@mail.sysu.edu.cn cxm@mail.sysu.edu.cn gaosong@mail.sysu.edu.cn

## Abstract

Celebrating the 100th Anniversary of Sun Yat-Sen University (SYSU), this themed collection features 29 recent cutting-edge articles and reviews in organic synthesis, coordination complexes, energy catalysis, functional materials, and biochemistry by SYSU scholars, alumni, and collaborators.
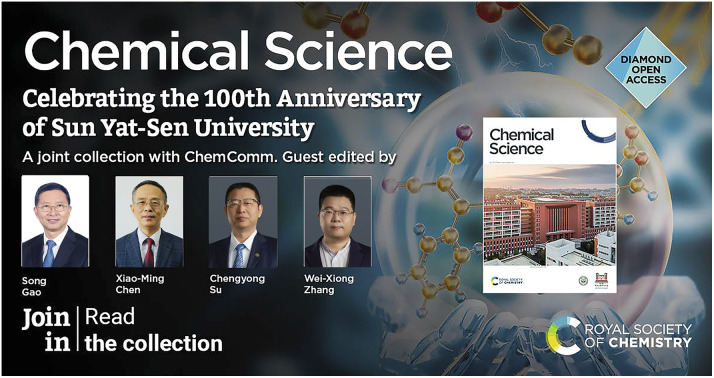

To develop education, the pioneer revolutionist Dr Sun Yat-Sen (also known as Zhongshan Sun) initiated the merger of several colleges and universities in Guangzhou, China, in February 1924, establishing the National Guangdong University. This institution was renamed the National Sun Yat-Sen University in August 1926 in honor of Dr Sun Yat-Sen, later also known as Zhongshan University and currently Sun Yat-Sen University (SYSU). Over the past century, SYSU has become a comprehensive research university driven by its motto “Study Extensively, Enquire Accurately, Reflect Carefully, Discriminate Clearly, and Practice Earnestly”. Inaugurated as the Department of Chemistry of the University in 1924, the current School of Chemistry at SYSU was established in 1994. Today, it comprises seven research institutes supported by world-class research faculty and state-of-the-art facilities.

Over the past decade, researchers from SYSU have published more than 400 articles in *Chemical Science* and *Chemical Communications*. To celebrate the 100th Anniversary of SYSU in 2024, the Royal Society of Chemistry has the pleasure of presenting this joint themed collection, bringing together 29 recent articles contributed by SYSU researchers, alumni and collaborators across diverse fields of chemistry. It is our great pleasure to introduce the articles from the collection in this editorial, spanning organic synthesis, coordination complexes, heterogeneous catalysis for energy applications, functional materials for devices, and applications of chemical biology.

## Design and synthesis of functional organic molecules

Kelong Zhu and coworkers designed a compact chemically driven [2]catenane rotary motor for precise 360° unidirectional rotation utilizing a molecular pumping system. The motor, propelled by an acid–base fuelled benzimidazolium pumping cassette, is recognized as the smallest catenane rotary motor to date (https://doi.org/10.1039/D4SC04292A). Zhongshu Li and coworkers successfully isolated and crystallographically characterized N-heterocyclic vinyl-substituted acyclic diphosphaenones. These compounds not only exhibit a central planar trans P=P–C=O configuration but also demonstrate conjugated 1,4-addition (https://doi.org/10.1039/D4SC06462C). Honggen Wang *et al.* introduced a unified stereodivergent synthetic method for synthesizing two isomers of alkene products, utilizing the same starting materials. This method allows for the utilization of various substituted alkyl iodides, facilitating easy access to both isomers of iodoalkene products bearing valuable functional groups at the allylic position (https://doi.org/10.1039/D4CC04948A). Zhuofeng Ke *et al.* presented a remote C–H bond cooperation strategy that enables the unprecedented homogeneous Ag(i)-catalyzed borrowing hydrogen/hydrogen auto-transfer reaction. They found that the strong electron-donating bis-*N*-heterocyclic carbene ligand can stabilize the silver-hydride and stimulates the hydride activity on the *trans*-position of ligands (https://doi.org/10.1039/D4SC05486E). Kuangbiao Liao *et al.* introduced SynAsk, a comprehensive large-language-model platform tailored for the organic chemistry domain, which can seamlessly access the knowledge base and advanced chemistry tools through a question-and-answer interface (https://doi.org/10.1039/D4SC04757E).

## Design and synthesis of functional coordination complexes

By investigating four coordination complexes based on different inorganic units [MCl_4_]^*n*−^ (M = Zn^2+^, Cd^2+^, Mn^2+^, or Sb^3+^), Xian-Ming Zhang and colleagues disclosed that electronic configurations of metal centres control the multi-mode emissions by manoeuvring the band alignments (https://doi.org/10.1039/D4SC05041J). To boost application of molecule-based ferroelectrics for self-powered piezoelectric sensors, La-Sheng Long and colleagues reported ferroelectric (Me_3_NCH_2_CH_2_Cl)[GaBr_4_] that exhibits the largest piezoelectric coefficient (*ca.* 454 pC N^−1^) among all the known free-standing polycrystalline pellets (https://doi.org/10.1039/D4SC04185B). They also synthesized another ferroelectric (Me_3_NCH_2_CH_2_Br)[GaBr_4_], exhibiting a piezoelectric coefficient of up to 331 pC N^−1^, and fabricated a composite piezoelectric sensor with a high power density (490 μW cm^−2^) enhanced by the synergistic combination of piezoelectric and triboelectric (https://doi.org/10.1039/D4SC05442C). Zhao-Ping Ni and colleagues reported two mononuclear iron(iii) complexes that demonstrate counterion dependence of their magnetic and fluorescence properties. They observed, for the first time, a synergistic effect between abrupt spin crossover and fluorescence in an iron(iii) complex (https://doi.org/10.1039/D4CC05036C). Jie-Peng Zhang and coworkers synthesized a novel SOD-topology metal azolate framework exhibiting halogen-bonding-controlled gating behaviour, which achieved an efficient purification of benzene from its binary and ternary mixtures with record selectivity of 113 ± 2 and a purity of over 98% (https://doi.org/10.1039/D4SC06624C). Ming-Hua Zeng *et al.* reported a “rivet” substitution and “hinge” linkage strategy for assembling the smaller [Zn_9_] subunits to a record-high [Zn_54_] nanocage, which aggregates to form a porous solid showing remarkable iodine vapour uptake capacity (https://doi.org/10.1039/D4SC04474F).

## Heterogeneous catalysis for environmental and energy applications

Gangfeng Ouyang and colleagues constructed a novel paired electrolysis system with a high overall energy efficiency of 34%, where fluorine-doped tin oxide glass serves as the anode for oxidizing water to H_2_O_2_ with a faradaic efficiency (FE) of 60%, and cobalt phthalocyanine/carbon nanotube loaded carbon paper as the cathode for reducing CO_2_ to CO with an FE exceeding 90% (https://doi.org/10.1039/D4CC04436C). Aiming at the photon-flux-density problem of sunlight for the photosynthesis of H_2_O_2_, Dan Li *et al.* designed several boron dipyrromethene-based cyclic trinuclear silver complexes to achieve photosynthesis of H_2_O_2_ with a record high production rate of 183.7 and 192.3 μM h^−1^ from pure water and sea water, respectively, without any additives (https://doi.org/10.1039/D4SC04098H). Guangqin Li *et al.* synthesized a series of rare-earth element doped NiFe-MOFs as efficient and robust bifunctional electrocatalysts. The representative CeNiFe-MOF showed a high oxygen-evolution-reaction performance benefiting from the optimized adsorption energy of H* and the reduced energy barrier from *OH to *O owing to Ce doping (https://doi.org/10.1039/D4SC06574C). Fei Xu *et al.* synthesized the Mo_2_C/MoS_2_ heterostructure on carbon paper (Mo_2_C/MoS_2_-CP), through rapid carbothermal shock in only two seconds, which exhibits superior intrinsic alkaline hydrogen-evolution-reaction activity and excellent stability for 100 h (https://doi.org/10.1039/D4CC03757J). Gangfeng Ouyang and colleagues developed novel oxygen-centred organic radicals with an impressive half-life of up to seven minutes in water, thus achieving highly efficient photocatalytic removal of organic pollutants even under an ultra-low light intensity of only 0.1 mW cm^−2^ (https://doi.org/10.1039/D4SC06339B).

## Functional materials for application in devices

Dingshan Yu *et al.* developed novel membrane electrolytes with high ionic conductivity (3.3 × 10^−4^ S cm^−1^) and Li^+^ transference number (0.74), endowing solid LiFePO_4_//Li batteries with excellent cyclability over 1000 cycles at 60 °C. Their strategy surmounts the ionic conduction-interface stability trade-off and thin dimension-flexibility conflict (https://doi.org/10.1039/D4CC04550E). Dingcai Wu and coworkers designed a super-structured multifunctional molecular brush and developed an ultrathin polymer electrolyte showing a remarkable capacity retention of 83% for Li/LiFePO_4_ cells at 1C after 1000 cycles. The solid-state fuel cell with a high-loading LiNi_0.8_Mn_0.1_Co_0.1_O_2_ cathode, delivers a high discharge specific capacity of 204 mA h g^−1^ for more than 400 cycles at a high cut-off voltage of 4.5 V (https://doi.org/10.1039/D4SC04454A). Yi Zhang and colleagues synthesized soluble photosensitive polyimide resins through the structural design of a bio-based magnolol monomer, and developed a polyimide photoresist that demonstrated impressive low-temperature curing performance at 180 °C. The resultant films exhibited high transparency with an average visible-light transmittance of 87.8% (https://doi.org/10.1039/D4SC07952C).

By integrating rigid π-extended donors with different acceptors, Zhenguo Chi and coworkers developed three highly-efficient blue thermally activated delayed fluorescence emitters, and they fabricated an organic light-emitting diode (OLED) exhibiting external quantum efficiency of 29.9% in the sky-blue region, along with low roll-off at high luminance (https://doi.org/10.1039/D4SC06613H). Mingmei Wu *et al.* reviewed the application of phosphor-converted LEDs in underwater optical communication and marine fishery, exploring the development strategies of phosphors tailored for the marine environment (https://doi.org/10.1039/D4SC06605G). Zhong-Ning Chen *et al.* strategically designed eco-friendly chiral manganese complexes with a circularly polarized luminescence asymmetric factor of 5.1 × 10^−3^, and fabricated a device revealing an external quantum efficiency exceeding 4% and an electroluminescent asymmetric factor reaching −8.5 × 10^−3^, surpassing most devices based on noble-metal complexes and perovskite related materials (https://doi.org/10.1039/D4SC04748F). By employing chain elongation and halogen substitution strategies, Dai-Bin Kuang *et al.* synthesized low-melting-point metal halides and obtained stable glass scintillators. The resulting glass exhibits a photoluminescence quantum yield of 47.6% and an impressive resolution of 25 lp mm^−1^ in X-ray imaging, and remains transparent even after being heated at 90 °C for six weeks (https://doi.org/10.1039/D4SC04229H). By optimizing the energy level alignment between the perovskite layer and the adjacent contacts, Wu-Qiang Wu *et al.* have successfully developed n–p homojunction-based perovskite solar cells without an electron transport layer, achieving an improved efficiency of *ca.* 13% higher than the control counterpart (https://doi.org/10.1039/D4CC05174B). Using scanning tunnelling microscopy break junction techniques, Jinghong Li *et al.* investigated the single molecule electrode coupling mode based on azulene controlled interface charge distribution, providing a new strategy for designing switchable single-molecule devices (https://doi.org/10.1039/D4SC06614F). Mei Pan *et al.* discovered an unexpected organic host–guest afterglow system, which was initiated by a paper (cellulose)-matrix approach and benefited from an efficient Förster resonance energy transfer from the host to the guest, thanks to the suitable host–guest energy level match (https://doi.org/10.1039/D4SC04746J).

## Biochemistry and nanodrugs

Liang Xu and colleagues integrated the host–guest recognition system into the core region of the 10–23 DNAzyme, and demonstrated that the function of DNAzyme could be reversibly and orthogonally controlled by utilizing cucurbit(7)uril and its competitive guests (https://doi.org/10.1039/D4CC05330C). Xiaogang Qu *et al.* identified a highly conserved and stable G-quadruplex (G4) in the STING promoter region, and further verify its function in transcriptional inhibition of STING using CRISPR technology to precisely target STING G4. Their findings may promote drug design for Alzheimer's disease (https://doi.org/10.1039/D4SC04453C). Jinsong Ren *et al.* constructed nanodrugs by structurally hybridizing an antioxidant defense pharmacological inhibitor and a co-nucleation precursor through the formation of carbon quantum dots. These nanodrugs exhibit dual-enhanced bioactivities, and they have a long body retention time and desirable biodistribution in living cells, enabling the elimination of melanoma cells at a very low injection dose (https://doi.org/10.1039/D4SC05280C).

We extend our best wishes to Sun Yat-Sen University on its 2024 centennial anniversary and hope our readers enjoy this exceptional collection.

